# The London County Westminster and Parr's Bank Ltd.

**Published:** 1921-01-15

**Authors:** 


### The London County Westminster and Parr's Bank Ltd.

A further dividend of 10 per cent. is declared in respect of the £20 shares, making 20 per cent. for the year, and a further dividend of 6¼ per cent. on the £1 shares will be paid, making the maximum of 12½ per cent. for the year.

